# Impact of the Charlson Comorbidity Index on Delirium Outcomes

**DOI:** 10.7759/cureus.70006

**Published:** 2024-09-23

**Authors:** Palanipriya Kalyan, Manisha Parulekar

**Affiliations:** 1 Department of Geriatrics, Hackensack University Medical Center, Hackensack, USA

**Keywords:** charlson comorbidity index, delirium, healthcare outcomes, inpatient, older adults

## Abstract

Introduction

Delirium is a significant inpatient healthcare challenge and has a greater incidence among older adults with adverse healthcare outcomes. Yet there are limited established models for risk stratification. The objective is to determine the effectiveness of implementing the Charlson comorbidity index (CCI) score, which is calculated based on the assigned weight for various disease conditions, and to analyze the healthcare outcomes in older hospitalized adults with delirium.

Methodology

A retrospective cohort study of 214 elderly hospitalized patients between January 1, 2015, and December 31, 2016, with the diagnosis of delirium, was analyzed by grouping based on the severity of diseases as defined in CCI. The primary analysis outcome was to analyze all-cause inpatient mortality, length of hospital stay (in days), 30-day readmissions, and discharge destination in patients with delirium based on CCI scores using regression analysis and nonparametric tests. Secondary analysis included the prevalence and characteristics of delirium patients in different severity levels of CCI.

Results

Patients with the severe CCI category (with a total score of five and above) spent 10 days longer in the hospital than those who were categorized with mild delirium (*p* = 0.011). There is a strong association between in-hospital mortality and the severe CCI category (odds ratio (OR), 4.566; 95% CI, 1.17- 1.86 (p = 0.035)). Also, patients with severe CCI scores were 4.6 times more likely to die during hospitalization compared to patients with less severe comorbidities. There were no significant differences found for discharge destination (OR, 0.702; 95% CI, 371- 1.328 (p = 0.277)) and readmission risk (OR, 1.660; 95% CI, 0.664- 4.149 (p = 0.278)) among different CCI groups.

Conclusions

Length of stay and inpatient mortality were significantly higher among the severe CCI category compared to the mild category. Our study suggests that CCI can help clinicians, patients, and their families in prognostication and better understanding of goals of care conversations.

## Introduction

Delirium has long been recognized as a risk factor for poor healthcare outcomes. It is associated with increased morbidity and mortality, a high rate of nursing home placement, and an increased risk for long-term cognitive impairment [1-4]. Several prior studies have identified various comorbid diseases as risk factors for developing delirium in older adults. Some of the comorbid conditions that have been analyzed previously include anemia, hypoxia, cardiac disease, chronic obstructive pulmonary disease, new-onset electrolyte imbalance, renal failure, vitamin D deficiency, history of atrial fibrillation, prior stroke/transient ischemic attack, history of neurologic comorbidity, peripheral artery disease, hypertension, prior cognitive impairment, history of carotid artery disease, development of acute kidney injury after surgical procedure, obstructive sleep apnea, sleep disorders, and lower serum level of albumin [5-10]. Other significant risk factors that are identified are old age, American Society of Anesthesiologists (ASA) physical status grade III, high body mass index, and history of alcohol excess. Frailty is an independent risk for delirium and is an important predictor of longer length of stay and discharge destination to a higher level of care [11, 12].

Assessment of activities of daily living (ADL) helps classify whether the patient can care for themse and be independent or dependent. The Katz Index of Independence in ADLs is one of the most commonly used tools to assess basic ADLs (bathing, dressing, toileting, transferring, continence, and feeding). Clinicians rate individuals as either fully independent (no supervision, direction, or personal assistance needed) or dependent (needing supervision, direction, personal assistance, or total care) across the above six skills, with a maximum score of six points indicating fully independent, four points for moderately impaired, and two points for severely impaired. This measure was originally created to assess the physical functioning among those who were in rehabilitation, and the grades of the index summarize overall performance. It has been developed to study the results of treatment and prognosis of illnesses in the elderly and chronically ill [13].

However, most of the previous studies examining the relationship between delirium and healthcare outcomes have not fully described the degree to which these comorbidities influence delirium outcomes. To detect patients who are at high risk for delirium early and to improve the outcome, it is vital to develop not only a strategy for risk stratification but also a risk assessment tool to help stratify outcomes once delirium is diagnosed. This study is specifically designed to assess patient outcomes using the Charlson Comorbidity Index (CCI) at the time of diagnosis of delirium. The CCI is the most widely used comorbidity index. It was originally developed to predict one-year mortality among 604 patients based on comorbidity data obtained from a hospital chart review in a single US hospital. The CCI score includes 19 different medical conditions, and each comorbid condition ranges from one to six points to sum an index score. In our study, patients with a total score of one to two were classified as mild CCI category, those with a total score of three to four as moderate CCI category, and those with a total score of five and above were classified as severe category [14, 15].

The hypothesis is that a higher CCI score would be associated with poor healthcare outcomes in delirious patients.

## Materials and methods

This retrospective study was conducted with all patients at and above age of 65 who were hospitalized in an academic medical center. Data were extracted directly from electronic health records for inpatient admissions between January 1, 2015, and December 31, 2016.

Medical comorbidities for each participant were extracted from the electronic health records by the presence of diagnosis codes identified using the International Classification of Diseases, Ninth Revision (ICD-9) 16], and International Classification of Diseases, Tenth Revision (ICD-10) 17]. Data were collected via a secure web form and were presented to the principal investigator as de-identified data in compliance with the Health Insurance Portability and Accountability Act (HIPAA). This study was approved and considered exempt by our hospital Institutional Review Board. 

Screening for cohorts was performed either by using the eligible diagnosis of delirium by the ICD-9 and ICD-10 code [16,17] or documentation of the positive Confusion Assessment Method (CAM) and the participants who met this screening criteria were included in the study. Patients admitted to inpatient palliative medicine/care units, psychiatry units, and inpatient hospices were excluded from the study. In addition, the following exclusion criteria were also applied: patient medical records with missing data on discharge status, mortality, and duplicate medical records showing more than one admission with delirium so that the patients were included in the sample only once during this study period.

Classification of comorbid conditions

Patients were classified according to the score calculated using various weighting categories of the CCI. Myocardial infarction, peripheral vascular disease, congestive heart failure, cerebrovascular disease/transient ischemic attack, dementia, chronic obstructive pulmonary disease (COPD), connective tissue disease, peptic ulcer disease, liver disease (mild: without portal hypertension), uncomplicated diabetes mellitus, each scored one. Diabetes mellitus with end-organ damage, hemiplegia, chronic kidney disease (severe: on dialysis, status post kidney transplant), uremia, moderate = creatinine >3 mg/dL (0.27 mmol/L), solid tumor without metastasis, leukemia, lymphoma, each scored two. Liver disease (severe: cirrhosis and portal hypertension with variceal bleeding history; moderate: cirrhosis and portal hypertension but no variceal bleeding history) scored three. Solid tumors with metastasis, and AIDS, each scored six [14].

For statistical analysis purposes, mild and moderate CCI categories were grouped together as CCI stage 1 and were compared with severe CCI category CCI stage 2. Prior studies had provided a theoretical justification that validated the use of such a summary of comorbidity measures when used in place of individual comorbidities [15].

The following additional variables were collected for each patient: age, gender, language, ethnicity, race, insurance status, living accommodation, sitter use, restraint order, hearing impairment, vision impairment, at-risk patients identified and followed by the Hospital Elder Life Program (HELP), and pre-existing dementia. Functional status was obtained using documented ADL for each subject [13]. In our study, this was collected to measure the functionality of the study population. Also, data on inpatient administration of high-risk medications was gathered, which had been defined using the Beers criteria that provided guidelines for safe and effective medication use while minimizing adverse drug events for the senior population. The “high-risk geriatric medications” included the following five categories of medications: anti-depressants, antipsychotics, benzodiazepines, sedatives, and opioids [18]. 

Outcomes

The primary aim was to analyze outcomes and measure all-cause inpatient mortality, length of hospital stay in days, 30-day readmissions, and discharge destination status in hospitalized geriatric patients with delirium. The secondary aim was to analyze the prevalence of delirium in different levels of CCI categories.

For statistical analysis, categorical variables were presented as counts and percentages using tests, and continuous variables were presented as means and standard deviations. The Mann-Whitney U test for nonparametric distribution of variable data was used.

Primary outcome was analyzed using logistic regression analysis and was performed to identify associations between variables, whereas linear regression was used for continuous measures. Modeled covariates in logistic regression analyses were reported as odds ratios (OR) with 95% confidence intervals (CI). A p-value < 0.05 had been chosen as the cut-off level for statistical significance. A binary logistic regression model was used to determine the association between CCI stage and health outcome. All data analysis was performed using IBM SPSS Statistics software, version 20.0 (IBM Corp., Armonk, NY).

## Results

Baseline demographics, medical history, and comorbidity characteristics of the study population classified by CCI stages are shown in Table 1 and Table 2, respectively.

**Table 1:** Baseline demographics of patients with delirium classified by the Charlson Comorbidity Index p<0.05, statistically significant

Characteristics no. (%)	Charlson Comorbidity Index Stage 1	Charlson Comorbidity Index Stage 2	p-value
	Mild to moderate (n=146)	Severe (n=68)	
Age, no. (%), years	78 (53.4)	32 (47.1)	0.38
65-80			
>80	68 (46.6)	36 (52.9)	
Gender, no. (%)	75 (51.4)	36 (52.9)	0.83
Men			
Women	71 (48.6)	32 (47.1)	
Language, no. (%)	103 (70.5)	35 (51.5)	0.007
English			
Non-English	43 (29.5)	33 (48.5)	
Ethnicity, no. (%)	115 (78.8)	43 (63.2)	0.01
White			
Non-White	31 (21.2)	25 (36.8)	
Race, no. (%)	107 (73.3)	40 (58.8)	0.102
White			
Black	8 (5.5)	4 (5.9)	
Asian	4 (2.7)	4 (5.9)	
Hispanic	11 (7.5)	4 (5.9)	
Other/ mixed	16 (11)	16 (23.5)	
Insurance status, no. (%)	7 (4.8)	2 (2.9)	0.03
Medicaid			
Medicare	116 (79.5)	45 (66.2)	
Other	23 (15.8)	21 (30.9)	
Living accommodation, no. (%)	98 (67.1)	42 (61.8)	0 .21
Home			
Elder care facility	17 (11.6)	14 (20.6)	
Unknown	31 (21.2)	12 (17.6)	
Activities of daily living, no. (%)	76 (52.1)	31 (45.6)	0 .55
Severely impaired			
Partially dependent/independent	25 (17.1)	11 (16.2)	
Unknown	45 (30.8)	26 (38.2)	
Mean length of hospital stay in days, Median (IQR)	8 (7)	6 (9)	0 .011

**Table 2:** Medical history and comorbidity characteristics of patients with delirium classified by the Charlson Comorbidity Index p<0.05, statistically significant

Characteristics	Charlson Comorbidity Index Stage 1	Charlson Comorbidity Index Stage 2	p-value
	Mild to moderate	Severe	
Delirium diagnosis	11 (7.5)	2 (2.9)	0.19
At admission			
During hospital stay	135 (92.5)	66 (97.1)	
Pre-existing dementia	100 (68.5)	22 (32.4)	0.00
Absent			
Present	46 (31.5)	46 (67.6)	
Hearing impairment	25 (17.1)	6 (8.8)	0.22
Present			
Absent	76 (52.1)	36 (52.9)	
Unknown	45 (30.8)	26 (38.2)	
Vision impairment	36 (24.7)	14 (20.6)	0.54
Present			
Absent	65 (44.5)	28 (41.2)	
Unknown	45 (30.8)	26 (38.2)	
Discharge destination for live patients	33 (24.4)	26 (39.4)	0.02
Home			
Higher level of care	102 (75.6)	40 (60.6)	
Mortality	11 (7.5)	2 (2.9)	0.19
Yes			
No	135 (92.5)	66 (97.1)	
30-day readmission	77 (57)	43 (65.2)	0.27
Yes			
No	58 (43)	23 (34.8)	
Potentially inappropriate medications (PIM)	9 (6.2)	3 (4.4)	0.43
Yes			
No	137 (93.8)	65 (95.6)	
Sitter requirement	9 (6.2)	3 (4.4)	0.51
Yes			
No	22 (15.1)	7 (10.3)	
No documentation	115 (78.8)	58 (85.3)	
Documented restraint use	0	0	0.25
Yes			
No	31 (21.2)	10 (14.7)	
Documentation unavailable	115 (78.8)	58 (85.3)	

Based on analyses of patient characteristics among 214 patients who were diagnosed with delirium, the mean age was 79.86 (SD, 8.3) years. A total of 103 patients (48.1%) had a CCI score of two or less, 43 patients (20.1%) had a CCI score of three to four, and 68 (31.8%) had a severity score of five or more in the Charleston comorbidity scale. Of the severity scores of five or more (31.8% group), four (1.9%) were highly morbid with a score of 10. Patients with severe comorbidities were more likely to be men (52.9% vs. 51.4%), age > 80 years (52.9% vs. 46.6%), and non-English-speaking group (48.5% vs. 29.5%) compared to patients with less severe diseases. Patients from elder care facilities had more severe comorbid illnesses (20.6% vs. 11.6%) compared to patients residing at home.

**Figure 1:**
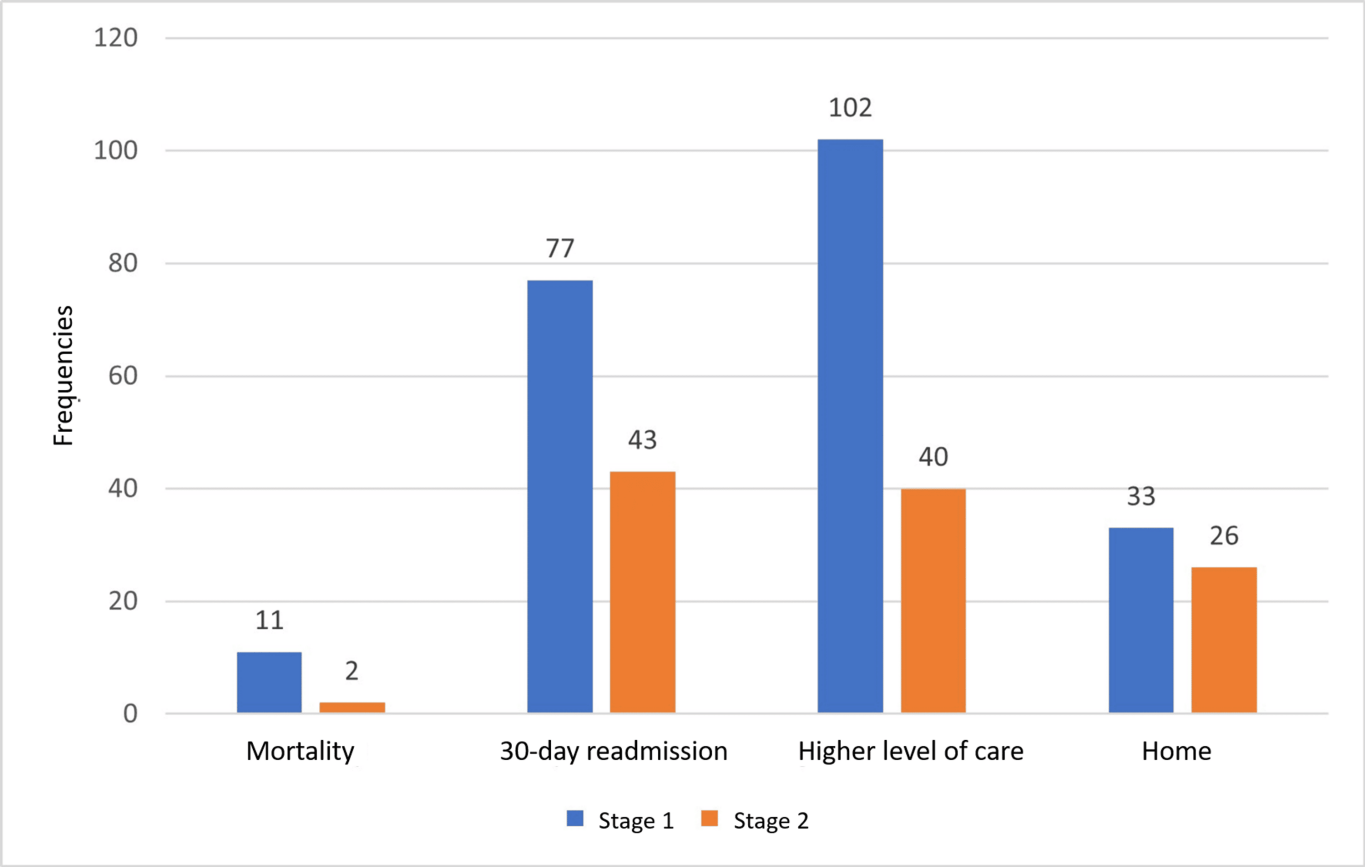
Distribution of outcome based on the Charlson Comorbidity Index stage category

Out of 214 patients, 140 (65.4%) were admitted from home, nine (4.2%) were transferred from other facilities such as skilled nursing, assisted living, or intermediate care facilities, and 22 (10.3%) from acute care facilities. Forty-three (20.1%) were admitted directly from the physician's office, and hence their living accommodation was not known. Among 201 patients, 59 (27.6%) were discharged to home, whereas 142 (66.4%) were to higher levels of care. The distribution of these outcomes among the two categories of CCI is shown in Figure 1.

Among patients discharged to higher levels of care, 21 (9.8%) were sent to acute rehabilitation centers, nine (4.2%) to long-term assisted living facilities, 86 (40.2%) to skilled nursing facilities, 15 (7.0%) to nursing homes, and 11 (5.1%) to hospice.

Premorbid functional status was assessed by the Katz index; 36 (16.8%) of patients were independent or mildly dependent, and 107 (50.0%) were severely or dependent in their daily functions. 

The average length of stay of the total study population was 10.29 days (SD, 10.93) in the hospital. The interquartile range (IQR) of hospital stay was found to be extensive (5.0-12.0), with a median length of seven days in hospital. Mann-Whitney U test showed that the length of inpatient stay was significantly higher in the severely comorbid group (Md = 8.00, n = 146) compared to the less severe comorbid group (Md = 6.00, n = 68), U = 3.90, Z = - 2.53, p = 0.011, with a medium effect size of r = 0.51.

Regression analysis after adjustment for baseline characteristics such as age, sex, ethnicity, language, and baseline dementia status showed that the comorbidity index was significantly associated with health outcomes as described in Table 3 and Table 4.

**Table 3:** Logistic regression summary of predictors of health outcome in delirious patients p<0.05, statistically significant; CCI: Charlson Comorbidity Index

Health outcome	Variable	Odds ratio (95% CI)	p-value
Discharge disposition	CCI stage 1	0.702 (0.37 – 1.32)	0.277
	CCI stage 2		
30-day readmission	CCI stage 1	1.660 (0.664 – 4.149)	0.278
	CCI stage 2		
Mortality	CCI stage 1	4.566 (1.17 – 1.86)	0.035
	CCI stage 2		

**Table 4:** Linear regression analysis summary for CCI severity predicting the length of stay R2 value = 0.58 ; p<0.05, statistically significant; CCI: Charlson Comorbidity Index

Variable	Regression coefficient	95% CI lower-upper	p-value
Age	1.082	0.072 - 0.182	0.02
Sex	2.497	0.367- 5.489	0.06
Language	.622	2.595-3.784	0.02
Ethnicity	0.138	0.044- 1.769	0.08
Pre-existing dementia	0.606	2.597-3.808	0.02
CCI severity score	1.808	1.132- 1.814	0.03

For CCI Stage 2, there was a strong association between mortality and CCI (OR, 4.566; 95% CI, 1.17- 1.86 (p = 0.035)). Patients with a higher CCI were 4.6 times more likely to die during hospitalization compared to patients with less severe comorbidities.

No association was found between patients discharge disposition and CCI (OR, 0.702; 95% CI, 371- 1.328 (p = 0.277)). Also, there was no association between 30-day readmissions and CCI (OR, 1.660; 95% CI, 0.664 - 4.149 (p = 0.278)).

Analysis using linear regression was used to assess whether the CCI score significantly predicted inpatient stay in patients with delirium. Results of the linear regression model after adjusting for baseline characteristics such as age, sex, ethnicity, language, and baseline dementia status showed that the CCI severity score explained 68% of the variance, R2 = 0.680, F(4,20) = 31.7, p = 0.015. Results showed that CCI severity predicted a longer length of stay, B = - 2.76, t = 1.72 (p < 0.05) in patients with delirium.

## Discussion

Based on the regression model, the CCI score of five or more was a strong predictor of healthcare outcomes such as mortality and longer length of hospital stay. The severe comorbidity index of inpatient hospitalized patients among patients with delirium was observed to be associated with a greater likelihood of inpatient mortality based on the OR. It did not show a statistically significant correlation between readmission rate or discharge destination.

In this retrospective analysis of 214 adults aged 65 and above who were identified as having delirium among hospital-wide admissions in one medical center, a high morbidity index was significantly associated with in-hospital mortality and length of stay but not associated with increased risk for 30-day readmission or placement to a high level of care.

This study is the first to our knowledge to investigate associations between delirium and health outcomes stratified by CCI. A unique aspect of this study is that the data included all medical comorbidities as incorporated in the CCI, allowing for an analytic design that has helped to compare patients based on the severity of the associated diseases. Results were robust to identify alternative methods of screening for poor outcomes in delirious populations.

Prior studies have shown analytic proof of the utility of comorbidity summary measures instead of individual comorbidities. Summary of comorbidity measures, such as the CCI and Elixhauser scores, are commonly used for clinical prognosis and comorbidity adjustment. A simulation study in 2015 has shown the utility of the summary comorbidity measures as substitutes for the use of individual comorbidity variables in health services research [19].

Interventions in minimizing high-risk medication use in managing older adults with multimorbidity have been widely discussed in the literature previously [20, 21]. These measures are undertaken for further accuracy of patient selection in our study.

A meta-analysis of 3,751 patients in 2015 suggests that multicomponent non-pharmacological interventions such as cognitive stimulation activities, orienting communication, early mobility, hearing, vision, hydration, and sleep-wake cycle preservation are effective in decreasing delirium incidence and preventing falls, potentially saving more than $16 billion annually in the United States alone. Therefore, these strategies hold great promise to influence two of the most important and prevalent conditions affecting seniors during hospitalization [22]. It also showed that length of stay and institutionalization trended toward decreases in the intervention groups, with a mean difference of -0.16 days shorter and the odds of institutionalization 5% lower than the non-intervention group. Our study suggests the importance of adding CCI in delirium risk stratification for both prevention and management strategies. The score can help target appropriate resources and interventions for high-risk populations.

Limitations

This study has several limitations that must be acknowledged. First, this analysis is not able to compare the health outcomes not attributable to delirium because nondelirious elderly hospitalized patients are not included in this study. Second, it has focused on the geriatric population and did not evaluate younger individuals at risk for delirium. Third, the generalizability of this study is limited because of the relatively few delirious patients with severe comorbidities in the study group. Lastly, given a smaller sample size, these outcomes are not able to be generalized, and further studies to confirm these findings are warranted.

## Conclusions

Among 214 geriatric hospitalized patients with multiple comorbidities, according to our study, delirium is observed to have a statistically significant association with prolonged hospital stay and inpatient mortality. Our study has shown that CCI scoring according to the severity of illnesses can help predict the risk of death and longer hospital stays among patients with delirium. These data may be helpful in prognosis and plan of care discussions with the families and caregivers of high-risk patients. However, further research is needed to better understand potential generalizability among younger and larger cohorts.

Recognizing the risk factors and diagnosing delirium promptly is important to improve the health outcome. Other measures of comorbidity, such as the CCI score in this study, should be considered to predict the outcome in delirious patients.
